# Supplementary material for: CD8 Co-Receptor Enhances T-Cell Activation without Any Effect on Initial Attachment
Source: Cells. 2021 Feb 18;10(2):429. doi: 10.3390/cells10020429 (PMC7922487; doi:10.3390/cells10020429)
Supplement: Supplementary file 1 [file cells-10-00429-s001.pdf]

Figure S1: Flow cytometry study of tested cells

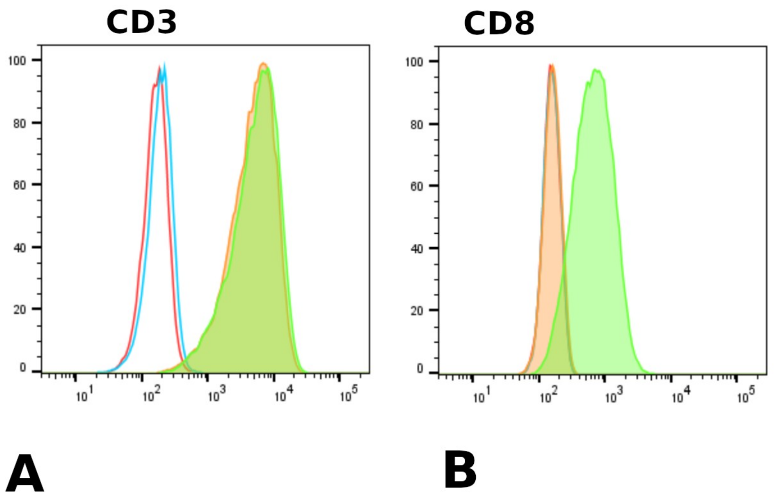

Cells used in our study were studied with flow cytometry. A – TCR-CD3 expression was studied on CD8+ (green line and area) and CD- (salmon line and area) cells. Isotype controls are also shown (white areas). B – CD8 expression was studied on CD8-transfected cells (green curve and area) and non-transfected cells (salmon line and area – fluorescence is the same as isotype controls).

TableS1: Results of binding experiments

Peptide	Mol / µm2	Stop CD8-/µm	Stop CD8+/µm	Surv 1s CD8-	Surv 1s CD8+	Surv 5s CD8-	Surv5sCD8+
3A	380.0	0.00079	0.00065	0.759	0.851	0.621	0.681
3A	190.0	0.00090	0.00143	0.841	0.797	0.794	0.662
3A	19.0	0.00070	0.00044	0.733	0.678	0.543	0.444
3A	9.5	0.00055	0.00060	0.692	0.587	0.5	0.347
3A	2.4	0.00069	0.00068	0.81	0.653	0.57	0.408
3Y	190.0	0.00056	0.00036	0.638	0.535	0.379	0.302
3Y	19.0	0.00005	0.00020	0.41	0.472	0.36	0.189
3Y	1.9	0.00004	0.00005	0.6	0.652	0.4	0.348
9L	380.0	0.00044	0.00074	0.733	0.763	0.584	0.639
9L	190.0	0.00066	0.00088	0.699	0.646	0.505	0.438
9L	19.0	0.00028	0.00030	0.868	0.58	0.714	0.348
9L	1.9	0.00004	0.00003	0.333	0.75	0.143	0.375
9V	1900.0	0.00069	0.00041	0.938	0.929	0.781	0.929
9V	190.0	0.00091	0.00064	0.795	0.882	0.628	0.737
9V	19.0	0.00032	0.00041	0.711	0.768	0.444	0.465
9V	1.9	0.00002	0.00004	1	0.5	0.6	0.25
H74	190.0	0.00035	0.00049	0.678	0.703	0.483	0.522
H74	19.0	0.00037	0.00055	0.652	0.765	0.411	0.587
H74	1.9	0.00019	0.00013	0.611	0.444	0.315	0.123

Results of binding measurements (5 pMHCs, 4 concentrations, 2 cell types) are shown. Data were used to perform overall sign tests.

Table S2 : Results of spreading studies

peptide	Mol / µm2	Lag CD8- s	Lag CD8+ s	Max slopeCD8-	Max slope CD8+	Area CD8- µm2	Area CD8+µm2	%SpreadCD8-	%SpreadCD8+
3A	23.75	165.6	157.8	0.66	1.38	6.3	38.1	30	60.7
3A	47.5	155.1	192.3	0.98	1.58	24.4	42.6	55	59.4
3A	95	159.7	193.7	2.56	3.21	41.4	43.6	63	66.8
3A	190	129.9	91.2	2.58	3.15	60.2	54.3	66	71.1
3Y	23.75	103.4	169.6	0.43	1.03	3.0	22.0	24.3	47.1
3Y	47.5	91.3	148	0.90	1.68	6.0	55.6	38.3	60.7
3Y	95	157	248.6	1.13	1.46	6.3	20.1	35.1	47.1
3Y	190	120	182.1	1.70	2.15	13.3	38.0	43.9	57.1
9L	23.75	148.3	122.8	1.32	0.75	4.7	10.4	23.5	37.1
9L	47.5	121.4	287.9	1.64	1.48	5.8	9.2	34.9	38.2
9L	95	186.6	194.5	1.26	1.61	7.4	13.3	34.8	50.4
9L	190	90.2	222.6	1.52	1.18	8.8	16.5	35.7	63.4
9V	23.75	127.6	141.3	1.40	2.09	13.8	44.3	53	63.9
9V	47.5	168.5	189.9	1.86	2.50	40.3	59.2	65	74.3
9V	95	94.7	63.8	2.63	2.10	59.0	59.5	72	72.2
9V	190	74.9	245.8	2.64	2.92	64.1	94.7	79	84.2
H74	23.75	35.4	236.1	1.29	1.53	26.1	33.3	60.6	59.4
H74	47.5	35.9	243.2	2.43	1.88	30.7	36.5	62.1	62.7
H74	95	56.4	164.9	3.02	3.08	42.5	32.6	69.1	61.5
H74	190	58.4	223.7	2.62	2.77	39.6	41.7	70.4	59.2

Results of spreading measurements (5 pMHCs, 4 concentrations, 2 cell types) are shown. Means were used to perform overall sign tests. Results obtained for each parameters combinations are shown on figures together with error bars.
